# Molecular, Phylogenetic and Immunohistochemical Detection of Bovine Mycoplasmosis in Clinical and Subclinical Mastitic Cows in Wasit Province, Iraq

**DOI:** 10.1002/vms3.70640

**Published:** 2025-09-30

**Authors:** Ahmed Jassim Almialy, Sattar J. J. AL‐Shaeli, Hasanain A. J. Gharban, Isra'a M. Essa

**Affiliations:** ^1^ Department of Veterinary Clinical Sciences Faculty of Veterinary Medicine University of Kufa Najaf Iraq; ^2^ Department of Medical Basic Sciences College of Dentistry University of Wasit Wasit Iraq; ^3^ Department of Internal and Preventive Veterinary Medicine College of Veterinary Medicine University of Wasit Wasit Iraq; ^4^ Department of Public Health College of Veterinary Medicine University of Basrah Basra Iraq

**Keywords:** histology, immunohistochemical, Iraq, *Mycoplasma bovis*, phylogenetic, tumour necrosis factor alpha

## Abstract

**Background:**

Bovine mycoplasmosis is a specific emerging infectious disease worldwide that causes clinical and subclinical mastitis, which impacts the global and national economy through reducing milk production and increasing treatment costs.

**Objective:**

The study detected the molecular prevalence of mycoplasmosis in clinical and subclinical mastitis infected lactating cows, phylogenic analysis of detected strain, immunohistochemical detection of inflammatory TNF‐α and identification of udder histological alterations.

**Materials and Methods:**

Udders of 263 lactating cows from various regions of Wasit Province, Iraq, were examined clinically, and subsequently milk samples were collected. California mastitis test (CMT) was performed to all clinical and subclinical milk samples, and the positive milk samples were examined molecularly by PCR to detect *Mycoplasma bovis* (*M. bovis)*. The phylogenic tree of positive samples was constructed using MEGA software based on obtained sequences from GenBank NCBI‐database. Furthermore, udder tissues were obtained to identify the immunohistochemical expression of TNF‐α and udder histological changes.

**Results:**

Clinical examination identified that 6.46% of study cows were infected with mastitis, whereas CMT identified 42.3% of subclinical mastitis cows. Molecular detection of *M. bovis* expressed positive prevalence rate in clinically and subclinically infected cows by 52.94% and 11.54%, respectively. Phylogenetically, the positive isolates showed sequences identical to the NCBI‐GenBank Egyptian *M. bovis* isolate with 99%–100% similarity and 0.0001%–0.0018% nucleotide changes. Histologically, the udder tissues exhibited a remarkable interstitial oedema, focal aggregation of inflammatory cells, infiltration of mononuclear cells, vacuolar degeneration, interstitial fibrosis, and destruction and desquamation of acinar epithelium. Immunohistochemically, the TNF‐α was expressed from mild to severe in udder tissues.

**Conclusion:**

The obtained results represent the unique study in Iraq that highlighted molecular detection of *M. bovis* in clinical and subclinical mastitic cows that was attributed to the Egyptian strain. However, specific quantitative and qualitative molecular and immunological studies that cover all Iraqi regions are required to identify the disease prevalence and economic losses that are caused by various bacterial pathogens, including *M. bovis* to provide precise management strategy to eliminate the disease.

## Graphical abstract

The prevalence of subclinical bovine mastitis is elevated in Iraq, which affected milk production. PCR detected *Mycoplasma bovis*, which is closely related to the Egyptian strain. Infected udders displayed histopathological changes and TNF‐α localization, which indicated significant tissue damage. This unique study highlighted the need for wide national surveys to quantify the impact and guide control strategies.

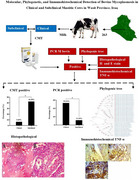

## 1 Introduction

Bovine mycoplasmosis, a complex and multifaceted disease affecting cattle, has garnered significant attention within the veterinary and scientific communities due to significant health and economic implications for livestock populations worldwide (Okella et al. 2023). Bovine mycoplasmosis is caused by a group of fastidious and pleomorphic bacteria belonging to the genus *Mycoplasma* that are classified under the Mollicutes family in the Mycoplasmatota phylum (Rostama and May 2021; Yan et al. 2024). This genus involves more than 126 species, which are considered the smallest living bacteria in nature and are characterized by lack of its cell wall and, therefore, highly resistance to β‐lactam antibiotics like penicillin (Gautier‐Bouchardon 2018; Ammar et al. 2021). In cattle, *Mycoplasma bovis* represents the main cause of mycoplasmosis and is known to colonize and infect various organs and systems. This infection is resulting in serious reproductive and respiratory dysfunction, otitis, arthritis and mastitis. These disorders lead to a major impediment to production due to reduced animal growth, a remarkable elevation of prevalence and death associated with massive economic decline (Priyantha et al. 2021; Garvey 2022; Kamel et al. 2024).

Mastitis is a prominent manifestation of bovine mycoplasmosis, which continues to be a significant health burden on the dairy industry primarily due to reduction in milk yield with high cost of treatment, impaired milk quality concomitant with specific treatment and culling (Nicholas et al. 2016; Mee et al. 2022). The prevalence of *M. bovis* as a clinically important species of *Mycoplasma* in dairy cows has reached to 21% (Maunsell et al. 2011; Dudek et al. 2020). After calving, although many infected cows displayed no clinical sign of mastitis and continuous producing milk that exhibited excessive levels of *M. bovis*, outbreaks of clinical *Mycoplasma* mastitis are often seen several weeks after an outbreak of respiratory disease (González and Wilson 2003; Fox 2012; Pothmann et al. 2015). However, the diagnosis of bovine mycoplasmosis can prove to be a complex effort, as the fastidious nature of the causative agents and the diverse range of clinical presentations can present significant challenges (Dudek et al. 2021; Gelgie et al. 2024). Recent advancements in diagnostic techniques such as molecular‐based assays have improved the ability to detect and identify the specific *Mycoplasma* species involved, enabling more targeted and effective treatment approaches (Gerace et al. 2022).

In Iraq, the prevalence of mastitis occurs in bovine animals, including cows and buffaloes in specific breeds that produce a high quantity of milk (Gharban and Yousif 2020; Wahab et al. 2024). The prevalence of clinical and, most importantly, subclinical mastitis can affect animal welfare and productivity, which consequently affects the national economy. These long term persistent challenges highlight the requirement of specific comprehensive strategies to address prevalence and diagnosis of *M. bovis* in both clinical and subclinical mastitis infections. Due to the absence of such studies in Iraq, therefore the objectives of the current study are to detect the prevalence of mastitis in dairy cows in clinical through routine examination and subclinical phases through the California mastitis test (CMT); furthermore, molecular testing of mycoplasmosis caused by *M. bovis* in mastitic animals is identified through PCR and detection of specific infected strains. Histological alterations of udder architecture concomitant with expression of TNF‐α were also identified through routine histopathological procedures and immunohistochemical techniques.

## 2 Materials and Methods

### 2.1 Clinical Examination and Samples Collection

A total of 263 lactating cows were randomly nominated from various regions, including rural and subrural small household farms from Wasit province (Iraq) during April to August 2024. Fundamentally, the udders of all study cows were examined clinically by a qualified professional expert veterinary doctor to detect the symptoms of clinical mastitis, including hotness, redness, rigidity and pain. After clinical assessment, approximately 100 mL of fresh milk were collected from each single quarter of the clinical and non‐clinical infected quarters of study cows under aseptic conditions post disposed initial milk stem from each quarter. Finally, the obtained milk samples were maintained in labelled plastic containers and transported to the laboratory under cooled conditions at 4°C using a small car refrigerator, and the samples were examined within 1–2 h after collecting.

### 2.2 California Mastitis Test

Essentially, the subclinical mastitic cases were identified through applying all collected milk samples to CMT examination using a specific kit (Kurdson Industry, Pakistan) as described by Saleem et al. (2021). The obtained data were classified into four scores based on the resulting gel clumping with changing the colour, which was interpreted as 0 (negative quarters), +1 (mild/weak positive), +2 (moderate positive), and +3; (severe/strong positive) (Fazal et al. 2023) as displayed in Figure S1. The cow was considered infected with mastitis if only a single quarter of the udder showed positive CMT.

### 2.3 Molecular Detection of *M. bovis*

According to the protocol of the Bacteria Kit (Presto Mini gDNA) from Geneaid (Taiwan), all positive clinical and subclinical milk samples were subjected to extraction of the DNAs, subsequently evaluated spectrophotometrically to measure the concentration and purity using the Thermo Scientific (UK) Nanodrop system. For the detection of the specific gene *16S rRNA*, the single primer set ([F: 5′‐ACT GAG ATA CGG CCC AGA CT‐3′] and [R: 5′‐TAC CGC TCC CAT GGT TTG AC‐3′]) was designed by Primer3Plus software and checked through Primer‐BLAST according to the NCBI‐GenBank using the *M. bovis* isolate (ID: AF332757.1). The MasterMix reaction was produced at 20 µL final volume in a PCR specific tube according to the instructions of the Accupower PCR PreMix Kit from Bioneer (Korea). PCR reaction was performed using the Bio Rad (USA) Thermal Cycler System to amplify target DNAs. The thermocycler condition was as follows: a single cycle at 95°C for 7 min initial denaturation, followed by 35 cycles at 95°C for 35 s final denaturation, at 54°C for 35 s annealing, at 72°C for 35 s initial extension, and single cycle at 72°C for 10 min final extension. The amplified DNAs were subjected to electrophoresis in Ethidium Bromide 1.5% Agarose‐gel (Biotech, Canada) at 100 V and 80 Am for 90 min. The PCR products were visualized under the UV Transilluminator (Clinx, China), and the positive samples were identified at approximately 1091 bp and photographed by the digital camera (Nikon, Japan).

### 2.4 Sequencing of PCR Product and Phylogenetic Analysis

The 21 PCR positive DNAs, including 9 clinical and 12 subclinical cases, were sequenced by the Macrogen Company (Korea). The sequenced data were submitted firstly to the NCBI‐GenBank database to obtain specific access numbers and then analysed phylogenetically to determine isolate identity with global NCBI‐BLAST *M. bovis* isolates/strains through using MEGA‐11 Software (Keklik 2023). The nucleotide sequences were first aligned using MAFFT v7.0 with default parameters to ensure high‐quality multiple sequence alignment. The best‐fit nucleotide substitution model was determined using ModelFinder implemented in MEGA 11 based on the Akaike Information Criterion corrected (AICc) and Bayesian Information Criterion (BIC). Among the tested models (JC, K2, Hasegawa–Kishino–Yano [HKY], TN93, GTR, and their variations with +I and +G), the HKY model (AICc = 2700.20) was selected as the most appropriate. Phylogenetic trees were constructed using the maximum likelihood (ML) method implemented in MEGA 11, with 1000 bootstrap replicates to evaluate the reliability of the branching topology. The resulting phylogenetic tree was visualized and annotated using Fig Tree v1.4.4.

### 2.5 Histology

Udder tissues of clinically mastitis positive cows were obtained post slaughter and processed histologically as described by Gharban et al. (2023). Briefly, the 10% neutral‐buffered formalin fixed tissues were dehydrated in elevated gradual concentrations of ethyl alcohol, cleared with xylol, infiltrated and embedded within a paraffin wax. The tissue was blocked and subsequently sectioned at approximately 5 µm of thickness, mounted on the slides using glycerol, and dried by air at room temperature. For staining, mounted tissue slides were subjected firstly to deparaffinization into lowered gradual concentrations of ethyl alcohol and then for staining using haematoxylin and eosin (BDH, England). The protocol of staining included immersion of slides into the jar containing haematoxylin solution for 30 s, rinsing in H_2_O for less than 1 min, immersion of slides into the jar containing 1% eosin solution for 10–30 s, dehydration with 100% ethyl alcohol for 30 s, and clearing with xylem. Finally, the slides are covered with the coverslip after glycerol dropping. Using the light microscope (MEIJI, Japan), the obtained slides were visualized and examined using ×100 and ×400 objective lenses.

### 2.6 Immunohistochemical Examination of Udders TNF‐α

The localization of TNF‐α in the udder tissues was detected immunohistochemically on the basis of methodology outlined by Gharban et al. (2023). The slides of histology were deparaffinized, rehydrated, and heated in 0.01 M citrate buffer for 30 min. Activity of endogenous peroxidase was blocked by H_2_O (3%) for 10 min, washed with PBS, and incubated at 4°C overnight with the anti‐bovine TNF‐α (Chemicon, Germany) as primary antibodies. Then, the sections were incubated at room temperature for 2 h with the diluted conjugated goat anti‐mouse as secondary antibodies and fluorescein isothiocyanate (FITC) for 2 h at room temperature. Finally, the slides were rinsed with PBS, mounted in fluorescent mounting medium and examined using light microscope (MEIJI, Japan) under an objective lens of ×100 and ×400. The intensity of staining was estimated following this designation: score (+1): 1%–20% of cells stained (mild), score (+2): 20%–50% of cells stained (moderate), and score (+3): >50% of cells stained (severe).

### 2.7 Statistical Analysis

The obtained data were stored and managed using Microsoft Excel 2019. The displayed data were applied to the GraphPad Prism Software (*version 8.0.1*) and subsequently analysed by *t*‐test to identify significant differences between the variables in both clinical and subclinical study groups and within them. The significant results were set (*) when *p* < 0.05.

## 3 Results

### 3.1 Clinical Evaluation of the Cow's Udders

**FIGURE 1:**
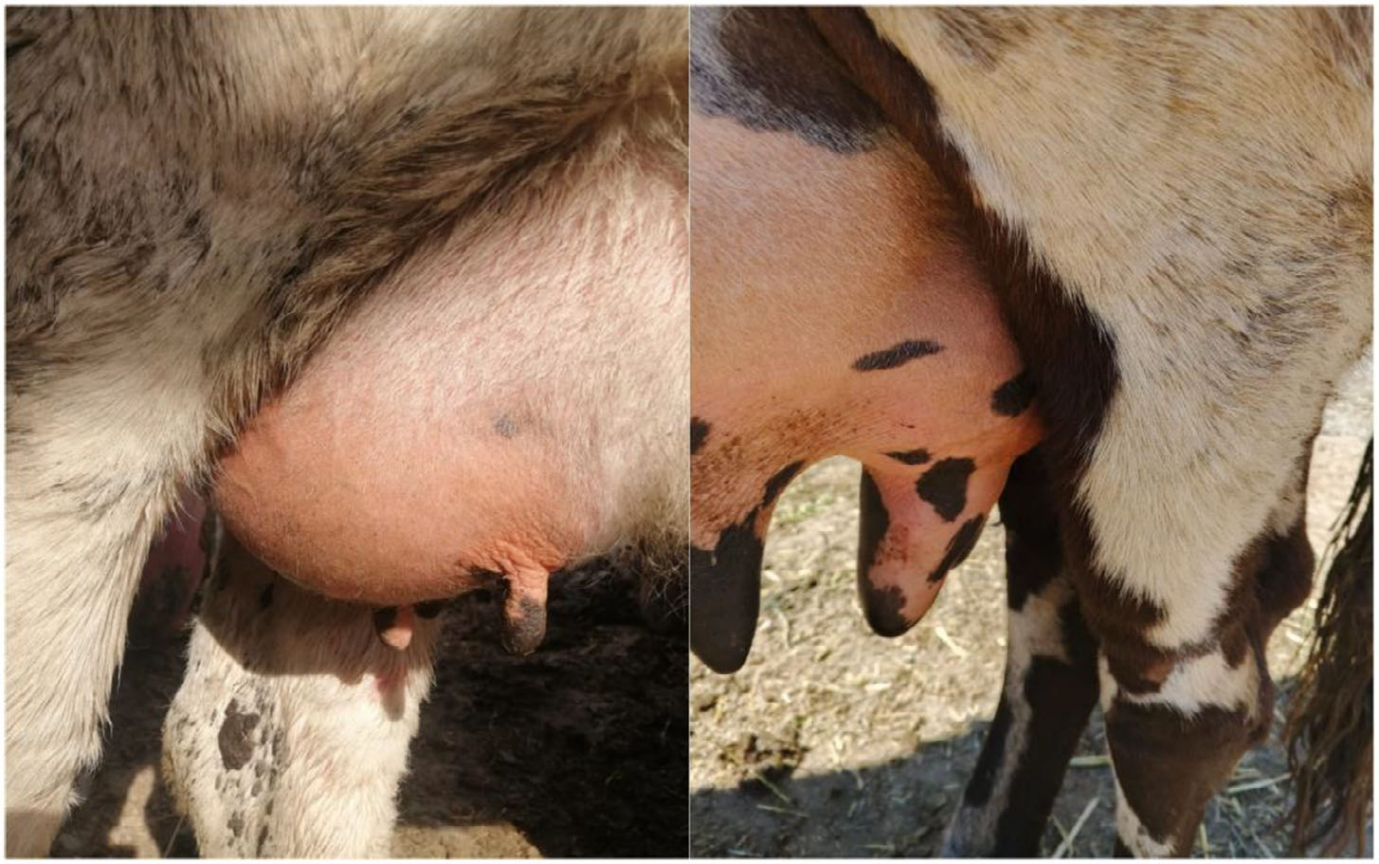
The signs of clinical mastitis infected cows.

**FIGURE 2:**
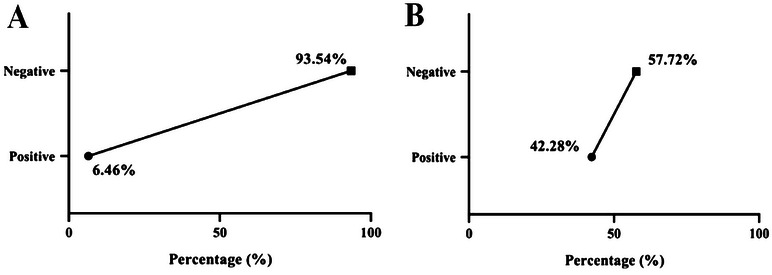
Clinical and subclinical mastitis infection in lactating cows. (A) Clinical positive mastitis among 263 lactating cows. (B) CMT subclinical positive mastitis among 246 non‐clinical infected cow.

Fundamentally, the clinical assessment of udders for an overall 263 lactating cows manifested obvious mastitis infection signs, including udder quarter swelling, tenderness, redness, hotness, and painfulness as seen in Figure 1. Furthermore, out of 263 examined cow, only 17 displayed signs of clinical mastitis infection, which represents 6.46%. Whereas, the remaining 246 cows showed no signs of mastitis infection, which represents 93.54% as displayed in Figure 2A.

### 3.2 CMT Subclinical Evaluation of Lactating Cow's Milk

**FIGURE 3:**
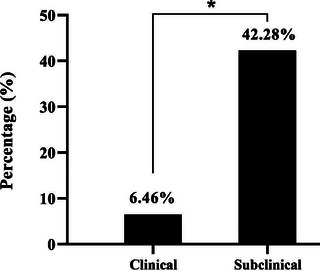
Clinical and subclinical positive mastitis infection in the study cows. (*P <0.05).

Interestingly, CMT testing showed that 104 cows were subclinically infected with mastitis out of 246 cows that expressed no signs of clinical mastitis, which represents 42.3% of positive results (Figure 2B). The remaining 142 cows were not infected with mastitis and represent 57.7% of negative results. Crucially, based on clinical and subclinical determined result of mastitis in dairy study cows, the prevalence of subclinical mastitis was significantly elevated (*p* < 0.0404, CI 203.2–251.9) compared to clinical mastitis prevalence (Figure 3).

### 3.3 PCR Evaluation of Positive Clinical and Subclinical Mastitis Milk

**FIGURE 4:**
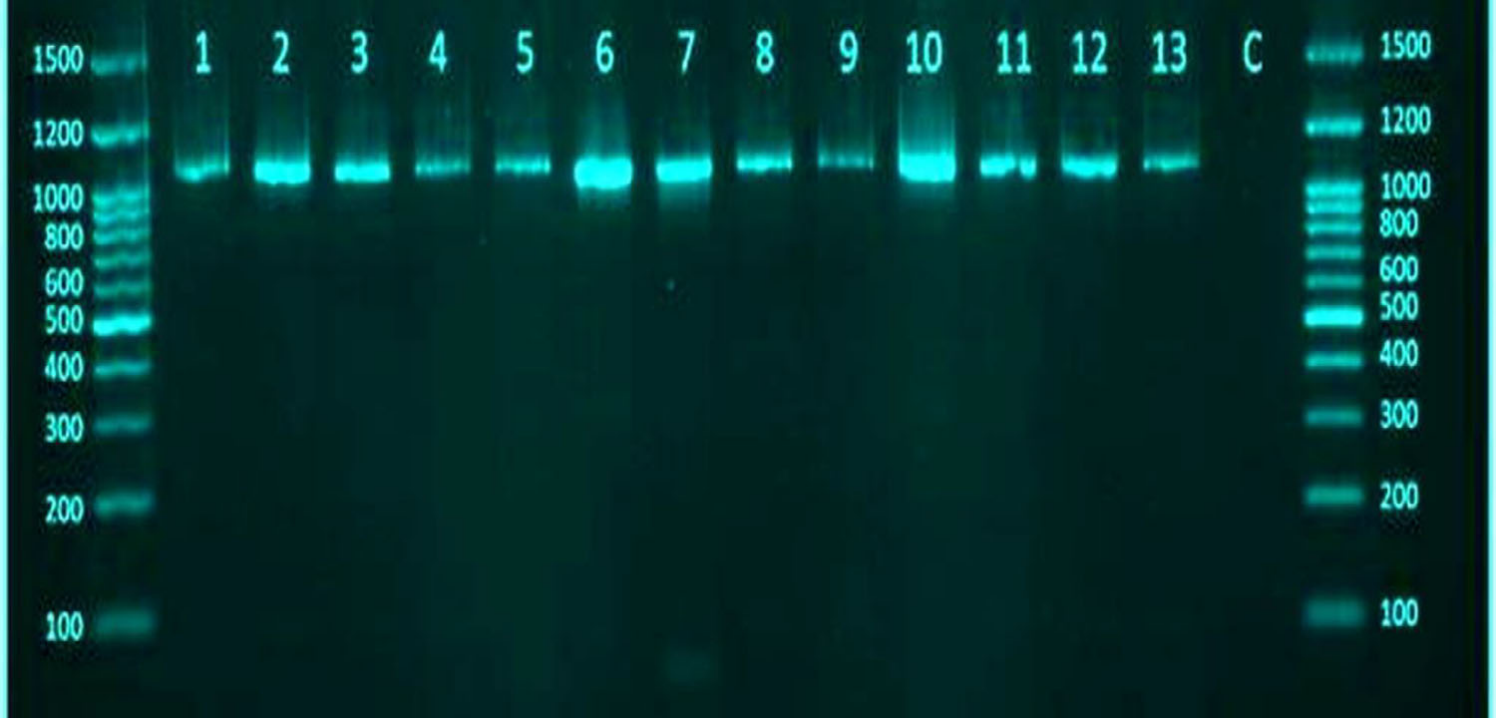
Agarose gel electrophoresis of positive PCR products. The Lane (M) corresponds to the ladder marker from 100 to 1500 bp, the lane (C) corresponds to the negative control, and lanes from 1 to13 are positive samples at 1091 bp.

**FIGURE 5:**
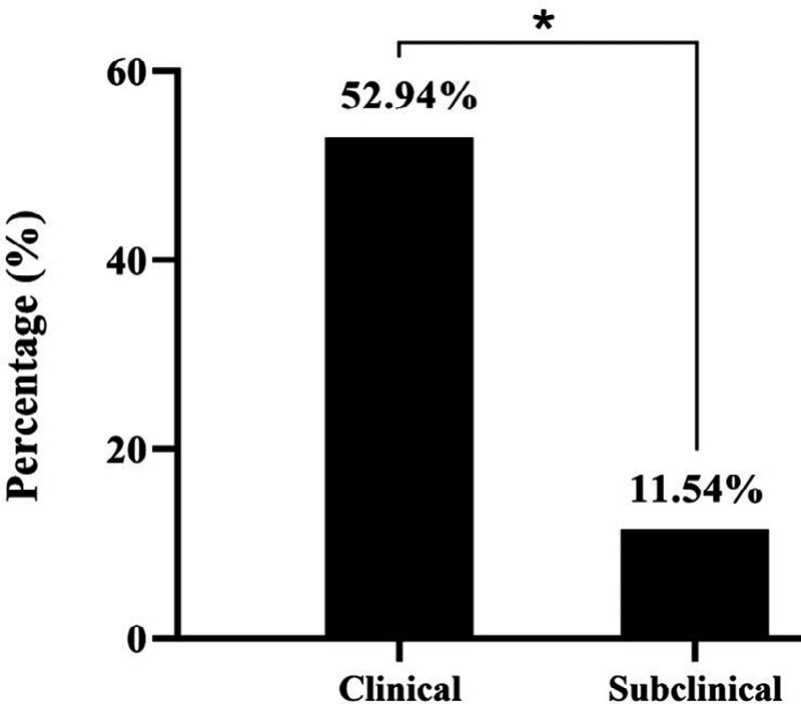
Molecular detection of *Mycoplasma bovis* in clinical and subclinical mastitis infected milk samples. (*P <0.05).

The milk samples of positive clinical (17) and subclinical (104) mastitis infected cows were molecularly detected by the conventional PCR assay (Figure 4). Interestingly, the result showed that 9 out of 17 (52.94%) and 12 out of 104 (11.54%) cows, respectively, were expressed positive specific gene for *M. bovis* (*p* < 0.0363, CI 230.8–295.3) (Figure 5).

### 3.4 Sequencing PCR Product and Phylogenetic Analysis

**FIGURE 6:**
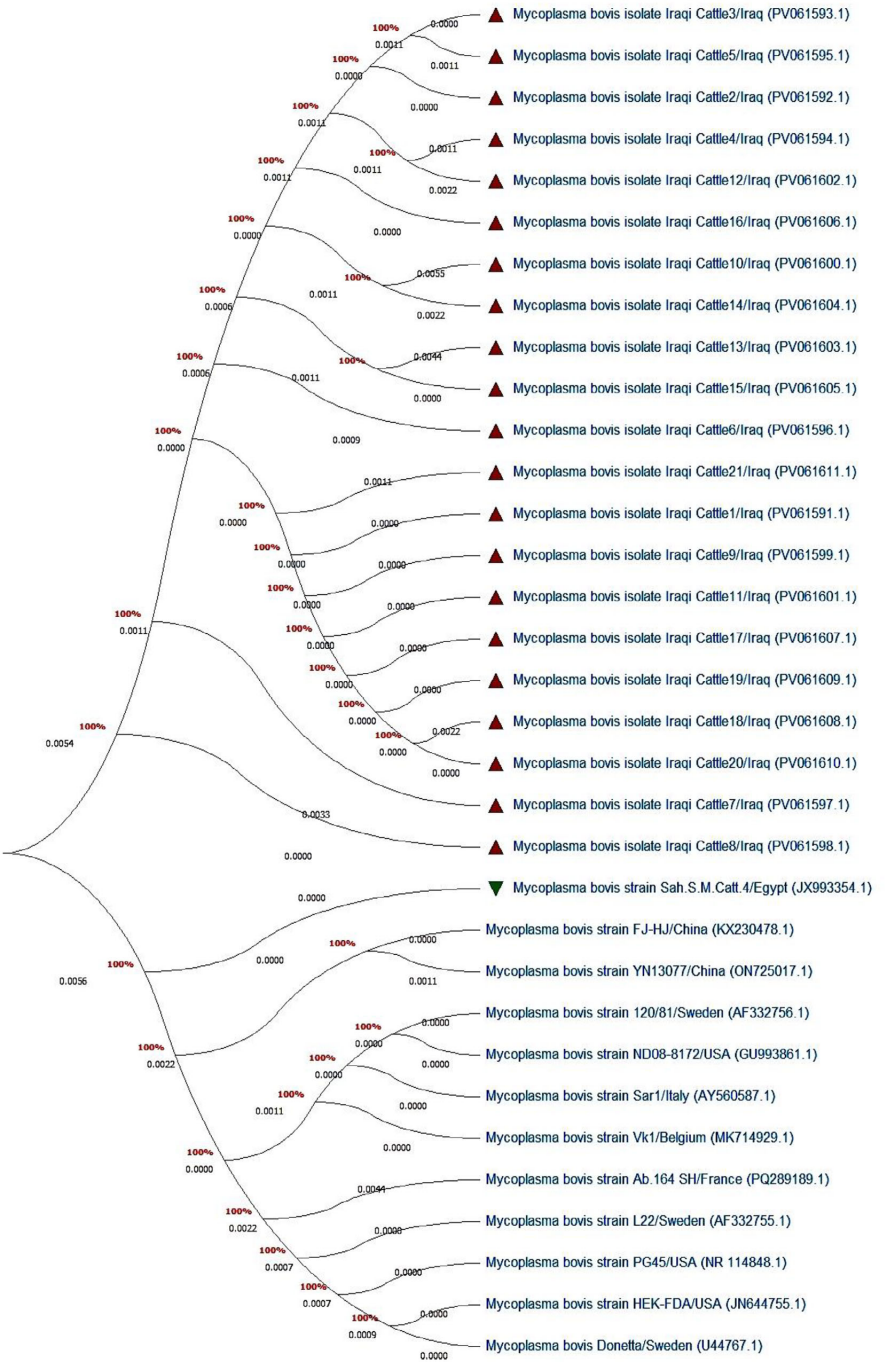
Phylogenetic tree analysis of *16srRNA* gene of *Mycoplasma bovis* local and NCBI‐BLAST isolates using the Maximum Likelihood (ML) method with Hasegawa–Kishino–Yano) HKY (model implemented in MEGA 11, with 1000 bootstrap replicates.

The sequencing data of 21 study isolates were submitted to the NCBI‐GenBank database under the names of Iraqi Cattle1‐21, and subsequently, their obtained IDs were PV061591–PV061611. The homology sequencing identity, multiple sequence alignment and phylogenic tree analysis of study isolates showed they were identical to the Egyptian *M. bovis* strain according to the global NCBI‐GenBank. The similarity ranged between 99% and 100%, and nucleotide changes between 0.0001% and 0.0018% (Table S1; Figure 6).

### 3.5 Histopathological Evaluation of Mastitis Cows Udders

**FIGURE 7:**
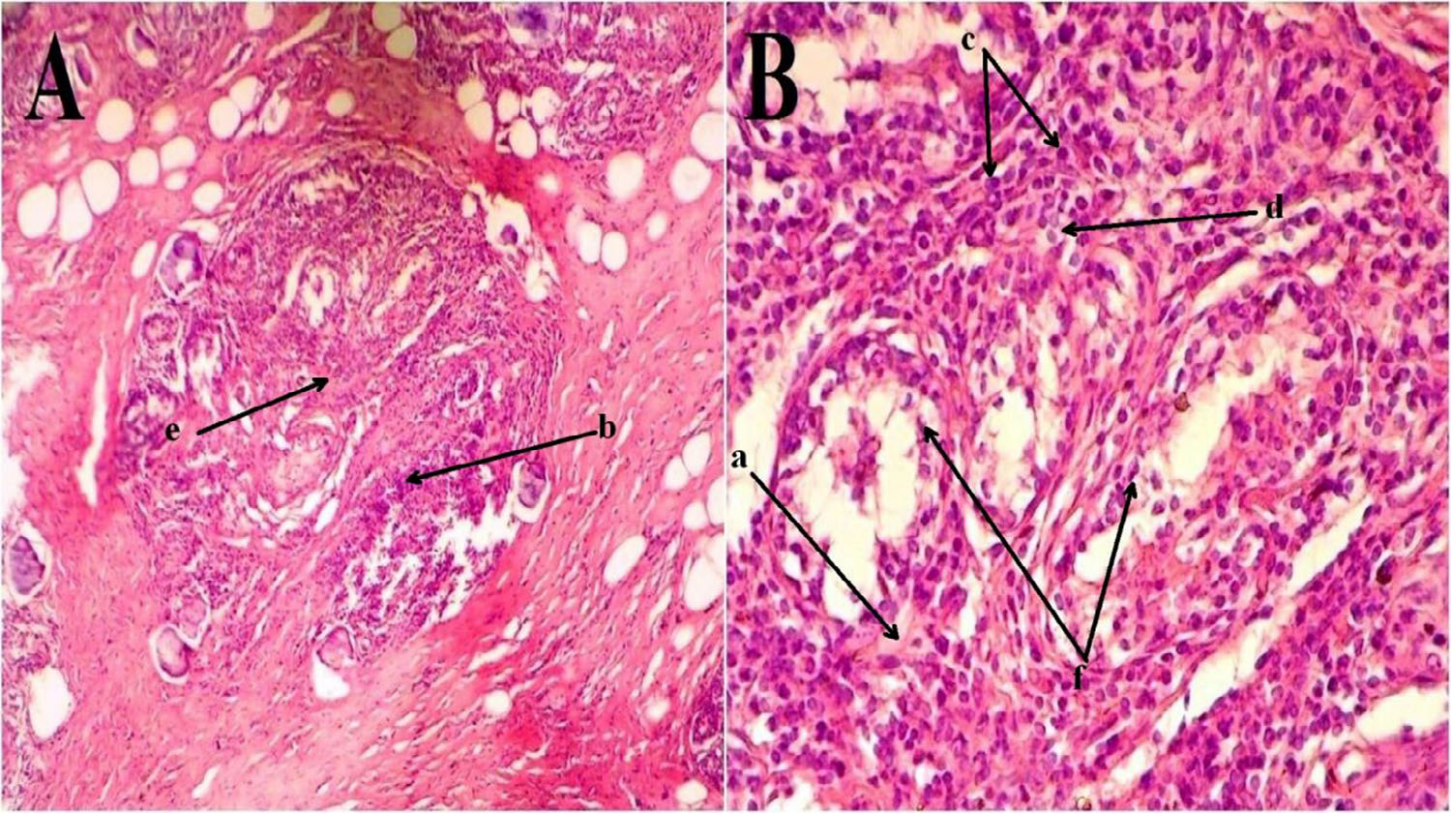
Histopathological changes of udder structure in mastitic cow. In both (A) (×100) and (B) (×400), various histopathological alterations can be seen. These changes include (a) interstitial oedema, (b) cellular inflammatory focal aggregation, (c) cellular mononuclear infiltration (neutrophils and macrophages) into the interlobular tissue, (d) vacuolar degeneration, (e) interstitial fibrosis, and (f) destruction and desquamation of acinar epithelium. The tissue was routinely processed and finally stained by Haematoxylin & Eosin stain.

Histopathological examination of clinically infected cow udder tissues showed a remarkable interstitial oedema, cellular inflammatory focal aggregation, cellular mononuclear infiltration (neutrophils and macrophages) into the interlobular tissue, vacuolar degeneration, interstitial fibrosis, and destruction and desquamation of acinar epithelium (Figure 7).

### 3.6 Immunohistochemical Evaluation of TNF‐α in Mastitis Cows Udders

**FIGURE 8:**
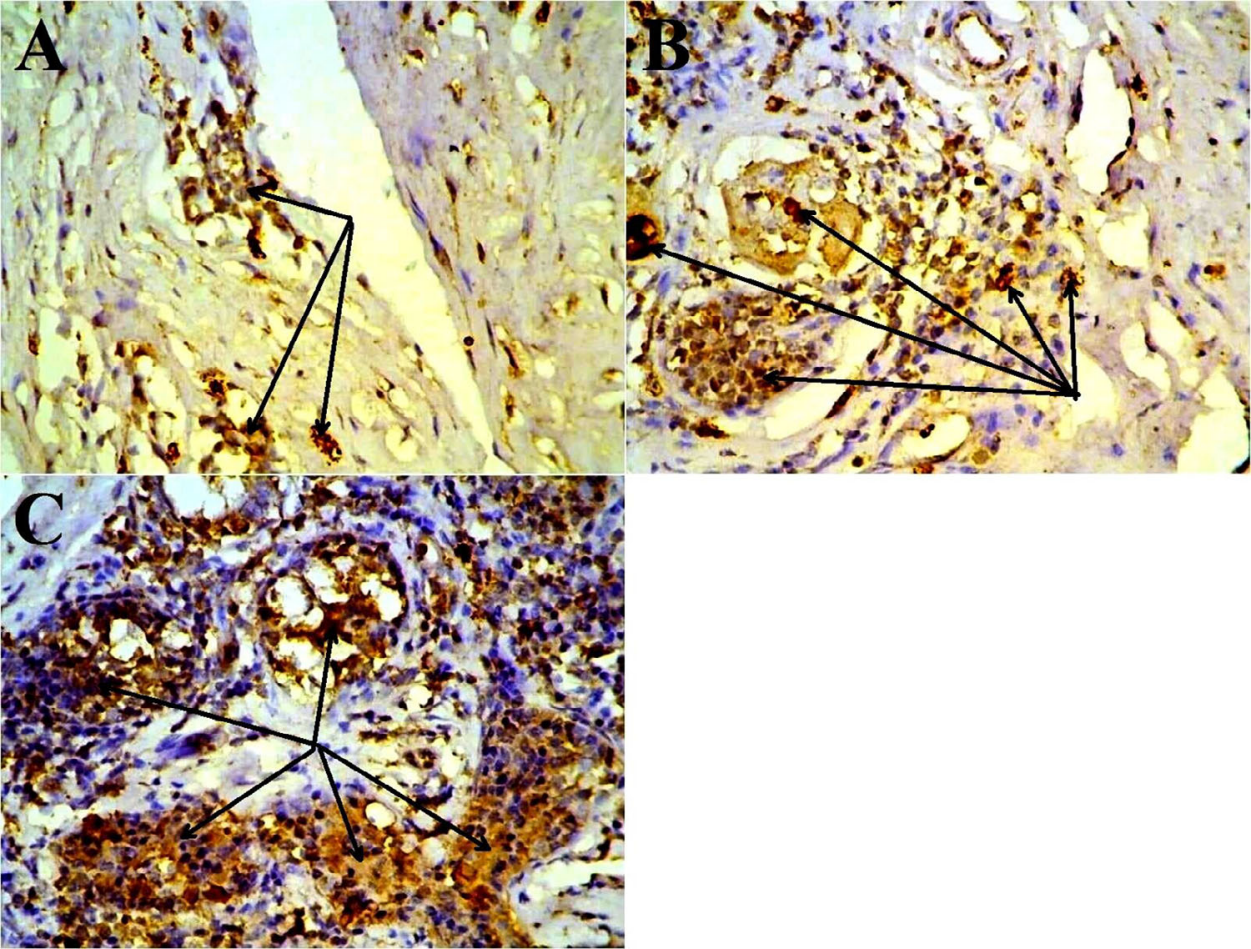
Immunohistochemical localization of TNF‐α in udder of mastitis cow ×400. The localization of TNF‐α was marked by arrows and ranging from mild (A), moderate (B), and strong (C).

Immunohistochemically, the localization of TNF‐α within the architecture of udder in cow clinically infected with mastitis was clearly seen in response to inflammatory status of the tissue. The intensity of TNF‐α expression was ranged from mild, moderate and severe as displayed in Figure 8.

## 4 Discussion

Mastitis is a major issue in lactating cows, particularly subclinical mastitis as the current study identified. Similarly, the prevalence of clinical (Minnat and Hammadi 2015; Jasim et al. 2022) and subclinical mastitis (Minnat and Hammadi 2015; Majeed 2016; Abdullah et al. 2017; Al‐Autaish et al. 2018; Al‐Bayati 2023) in cows due to various etiologies has been estimated in various regions in Iraq. In line with these studies, the current study showed significant prevalence of subclinical mastitis in comparison with clinical prevalence. The same data were reported nationally by Minnat and Hammadi (2015) as they recorded 32% for clinical and 68% for subclinical mastitis, and internationally by Argaw (2016), who mentioned that the high economic decline in bovine dairy herds is attributed to a highly subclinical mastitis prevalence than the clinical cases. According to territories distribution analysis of subclinical mastitis, the high prevalence was recorded in North America by 46%, then Africa by 44%, Asia comes third by 42%, followed by Europe and Oceania at 37% and 36%, and lastly Latin America by 34% (Krishnamoorthy et al. 2021). Whereas, the global prevalence of clinical mastitis is estimated at 15%, with a high prevalence (29%) in Europe and lowest prevalence (5%) in Oceania (Krishnamoorthy et al. 2021). However, cases of mastitis in the field have been attributed to inadequate hygiene during milking, excessive suckling of the newborn, and contact with contaminated pastures (Bekuma and Galmessa 2018). Other authors reported possible causes of mastitis prevalence could be attributed to livestock farming perception due to greenhouse adverse impact, milk loads cross contamination, improper genetic husbandry that produced dairy cattle with high udder susceptibility to infection, ageing cow decline in udder immunity, and inadequate implemented strategies for prevention and control of mastitis prevalence in dairy farms (Zadoks and Fitzpatrick 2009; Gupta and Hussain 2018; Krishnamoorthy et al. 2021).

The pathogens that cause mastitis can be divided on the basis of epidemiological factors into contagious, including *Streptococcus uberis*, *Staphylococcus aureus*, coagulase‐negative *Staphylococci Streptococcus agalactiae*, and *M. bovis*, and environmental, including *Escherichia coli*, *Klebsiella* spp., *Streptococcus dysgalactiae*, and *Trueperella pyogenes* (Constable et al. 2016). Isolation of *Mycoplasma* species, particularly *M. bovis*, from clinical and subclinical mastitis has been done previously (Al‐Farha et al. 2017; Hazelton et al. 2020). In this study, molecular examination targeting *M. bovis* in milk samples showed the high prevalence of infection in the udders of clinically infected cows (52.94%) when compared to subclinical mastitis (11.54%). In Iraq, the several specific studies have been investigated bovine mastitis and identified various pathogens such as *S. aureus* and Coagulase‐negative staphylococci (*S. sciuri*, *S. lentus*, *S. gallinarum*, *S. warneri*, *S. xylosus*, *S. lugdunensis*, *S. haemolyticus*, *S. cohnii*, *S. hyicus*, *S. saprophyticus*, *S. intermedius* and *S. epidermidis*) (Mahmmoud 2006; Al‐Jumaily et al. 2012; Abed and Hamim 2015; Al‐Hamdani and Al‐Ghanimi 2016); *Micrococcus* spp. (Yass and Judi 1996); *Streptococcus* spp. (*S. agalactiae*, *S. dysgalactiae*, and *S. uberis*), (Hussein 2012; Al‐kuzaay and Kshash 2013); *Bacillus cereus* (Yousif et al. 2008; Abdulla et al. 2011); *E. coli* (Kshash et al. 2009); *Klebsiella pneumoniae* (Nadhom 2018); *Salmonella* spp. (*S. typhimurium*), (Hassan 2012); *Pseudomonas aeruginosa* (AL‐Taee et al. 2019); *Serratia* spp. (*S. marcescens*), (Abdullah et al. 2017); *Proteus* spp. (Majeed 2016); and *Pasteurella multocida*/*P. heamolytica* (Yousif et al. 2008). According to the pervious wide Iraqi studies that detected mastitis in bovine, the aim and result of the current study are unique due to detected and confirmed the incidence of *M. bovis* in clinical and subclinical mastitic cows. In line with the current study result, the global *M. bovis* prevalence in the cattle infected with mastitis is 8.2% in Northern Greece (Filioussis et al. 2007), 21.11% in Turkey (Karahan et al. 2010), 2%–2.4% and 51.5% in Egypt (Ouda et al. 2016; Ahmed et al. 2023), 6.2% in Australia (Al‐Farha et al. 2017), and 3% in Brazil (Junqueira et al. 2020).

Importantly, construction of phylogenetic tree of isolated samples in the current study detected close relation with *M. bovis* Egyptian strain (Ahmed et al. 2023). This result suggests a complex interplay of factors, including pathogen dissemination mechanisms, host‐pathogen interactions, and potentially convergent evolutionary pressures, which might contribute to broad distribution and genetic stability across different regions (Olaogun et al. 2015; Sironi et al. 2015). In addition, the absence of rigorous animal health management programmes further contributes to unrestricted movement of infected animals and maintenance of disease prevalence across national borders (Wilhite 2019; Clemmons et al. 2021). The prevalence of *Mycoplasma* spp. as a causative agent of mastitis, coupled with limited diagnostic and control measures in many developing dairy industries, could exacerbate the global spread and genetic uniformity of *M. bovis* (Gelgie et al. 2024).

In the current study, different histopathological characteristics of acute and diffused interstitial lesions have been seen throughout the udder tissues, indicating severe tissue damage induced by the bacterial infection, *M. bovis*. These changes were similar with that observed by other studies in large (Bianchi et al. 2019; Chang et al. 2019) and small (EZ Kotb et al. 2020; Al‐Graibawi and Yousif 2021; Arteche‐Villasol et al. 2022) ruminants. According to the results of the previous studies that investigated several various tissues with pathological states, the current study concluded that there are dramatic alterations in a variety of immunological factors, including TNF‐α, which likely increased in response to mastitis and thus could possess a particularly important role in the progression of infection and recovery processes. Targeting the TNF‐localization in the udder tissue through immunohistochemical showed mild, moderate and severe scores of infection. This might be attributed to severity of disease and the concentration of pathogen in the target tissue, which consequently stimulated local and systemic status of inflammation and thus released several inflammatory mediators, especially TNF‐α. The latter is expressed in udder tissue due to a local inflammatory process that could be extended to the circulation as systemic inflammatory stimulation (Ingman et al. 2014; Cheng and Han 2020).

## 5 Conclusion

The current study detected that the subclinical prevalence of bovine mastitis has remarkably risen, which affected management and economy. Interestingly, *M. bovis* is molecularly detected as a causative agent that caused clinical and subclinical mastitis in Wasit province, Iraq. Sequencing and phylogenetic tree construction of study isolates showed close relation with the Egyptian *M. bovis* strain. These results marked alteration of infected udder tissue concomitant with immunohistochemical localization of TNF‐α, making the current study first and unique Iraqi investigation. The current study result, with pervious global and national studies results, highlighted the negative impact of mastitis, in clinical, and most importantly, subclinical, on ruminant farming fields and crucially involved in overall national and global economic decline. Therefore, wide and specific molecular phylogenic studies that investigate all aspects of ruminant mastitis epidemiology and risk factors in Iraq are required to build an accurate map of ruminant mastitis epidemiology and causes and also to highlight the awareness to increase routine detection of mastitis, particularly in subclinical infection.

## Author Contributions

**Ahmed Jassim Almialy**: molecular examination of milk samples. **Sattar J. J. AL‐Shaeli**: Histological and immunohistochemical processing. **Hasanain A. J. Gharban**: clinical examination, collection of milk samples, and phylogenetic analysis. **Isra'a M. Essa**: statistical analysis. All authors contributed equally and approved the final copy of the manuscript.

## Ethics Statement

The authors reported that the current study was performed in accordance with College of Veterinary Medicine (University of Wasit) animal care legislation and Scientific Committee was approved the ethics of this work under license No. 51‐16/3/2024.

## Conflicts of Interest

The authors declare no conflicts of interest.

## Peer Review

The peer review history for this article is available at https://www.webofscience.com/api/gateway/wos/peer‐review/10.1002/vms3.70640.

## Supporting information




**FIGURE S1**: Reaction of examined milk based on CMT kit.
TABLE S1 Homologous identity sequence (%) of local with NCBI‐BLAST strains.

## Data Availability

The data that support the findings of this study are available from the corresponding author upon reasonable request.
