# Supplementary material for: Molecular, Phylogenetic and Immunohistochemical Detection of Bovine Mycoplasmosis in Clinical and Subclinical Mastitic Cows in Wasit Province, Iraq
Source: Vet Med Sci. 2025 Sep 30;11(6):e70640. doi: 10.1002/vms3.70640 (PMC12482515; doi:10.1002/vms3.70640)
Supplement: Supplementary file 1 — FIGURE S1: Reaction of examined milk based on CMT kit. TABLE S1 Homologous identity sequence (%) of local with NCBI‐BLAST strains. [file VMS3-11-e70640-s001.docx]

**Supplementary figures and tables**

**Figure (1): Reaction of examined milk based on CMT kit**

**Table (1): Homologous identity sequence (%) of local with NCBI-BLAST strains**

| **Local isolate** | | **NCBI isolate** | | | |
| --- | --- | --- | --- | --- | --- |
| **Name** | **Access No.** | **Species** | **Country** | **Access No.** | **Homolog %** |
| Iraqi Cattle1 | PV061591.1 | *M. bovis* | Egypt | JX993354.1 | 100 |
| Iraqi Cattle2 | PV061592.1 | *M. bovis* | Egypt | JX993354.1 | 100 |
| Iraqi Cattle3 | PV061593.1 | *M. bovis* | Egypt | JX993354.1 | 99 |
| Iraqi Cattle4 | PV061594.1 | *M. bovis* | Egypt | JX993354.1 | 99 |
| Iraqi Cattle5 | PV061595.1 | *M. bovis* | Egypt | JX993354.1 | 99 |
| Iraqi Cattle6 | PV061596 | *M. bovis* | Egypt | JX993354.1 | 100 |
| Iraqi Cattle7 | PV061597.1 | *M. bovis* | Egypt | JX993354.1 | 99.78 |
| Iraqi Cattle8 | PV061598.1 | *M. bovis* | Egypt | JX993354.1 | 100 |
| Iraqi Cattle9 | PV061599.1 | *M. bovis* | Egypt | JX993354.1 | 99.43 |
| Iraqi Cattle10 | PV061600.1 | *M. bovis* | Egypt | JX993354.1 | 99.56 |
| Iraqi Cattle11 | PV061601.1 | *M. bovis* | Egypt | JX993354.1 | 99.34 |
| Iraqi Cattle12 | PV061602.1 | *M. bovis* | Egypt | JX993354.1 | 99.78 |
| Iraqi Cattle13 | PV061603.1 | *M. bovis* | Egypt | JX993354.1 | 99.89 |
| Iraqi Cattle14 | PV061604.1 | *M. bovis* | Egypt | JX993354.1 | 99.89 |
| Iraqi Cattle15 | PV061605.1 | *M. bovis* | Egypt | JX993354.1 | 100 |
| Iraqi Cattle16 | PV061606.1 | *M. bovis* | Egypt | JX993354.1 | 100 |
| Iraqi Cattle17 | PV061607.1 | *M. bovis* | Egypt | JX993354.1 | 99.34 |
| Iraqi Cattle18 | PV061608.1 | *M. bovis* | Egypt | JX993354.1 | 99.78 |
| Iraqi Cattle19 | PV061609.1 | *M. bovis* | Egypt | JX993354.1 | 99.13 |
| Iraqi Cattle20 | PV061610.1 | *M. bovis* | Egypt | JX993354.1 | 99.97 |
| Iraqi Cattle21 | PV061611.1 | *M. bovis* | Egypt | JX993354.1 | 99.45 |
